# Framing and self-responsibility modulate brain activities in decision escalation

**DOI:** 10.1186/s12868-021-00625-4

**Published:** 2021-03-23

**Authors:** Ting-Peng Liang, Yu-Wen Li, Nai-Shing Yen, Ofir Turel, Sen-Mou Hsu

**Affiliations:** 1 Electronic Commerce Research Center, National Sun Yat-Sen University, 70 Lienhai Rd., Kaohsiung, 80424 Taiwan; 2 Department of Digital Content Application and Management, Wenzao Ursuline University of Languages, Kaohsiung, Taiwan; 3 Department of Psychology, National Chengchi University, Taipei, Taiwan; 4 School of Business and Economics, California State University, Fullerton, USA; 5 Image Center for Integrated Body, Mind, and Culture Research, National Taiwan University, Taipei, Taiwan

**Keywords:** Escalation of commitment, Framing effect, Responsibility, Functional magnetic resonance imaging (fMRI)

## Abstract

**Background:**

Escalation of commitment is a common bias in human decision making. The present study examined (1) differences in neural recruitment for escalation and de-escalation decisions of prior investments, and (2) how the activations of these brain networks are affected by two factors that can arguably modulate escalation decisions: (i) self-responsibility, and (ii) framing of the success probabilities.

**Results:**

Imaging data were obtained from functional magnetic resonance imaging (fMRI) applied to 29 participants. A whole-brain analysis was conducted to compare brain activations between conditions. ROI analysis, then, was used to examine if these significant activations were modulated by two contextual factors. Finally, mediation analysis was applied to explore how the contextual factors affect escalation decisions through brain activations. The findings showed that (1) escalation decisions are faster than de-escalation decisions, (2) the corresponding network of brain regions recruited for escalation (anterior cingulate cortex, insula and precuneus) decisions differs from this recruited for de-escalation decisions (inferior and superior frontal gyri), (3) the switch from escalation to de-escalation is primarily frontal gyri dependent, and (4) activation in the anterior cingulate cortex, insula and precuneus were further increased in escalation decisions, when the outcome probabilities of the follow-up investment were positively framed; and activation in the inferior and superior frontal gyri in de-escalation decisions were increased when the outcome probabilities were negatively framed.

**Conclusions:**

Escalation and de-escalation decisions recruit different brain regions. Framing of possible outcomes as negative leads to escalation decisions through recruitment of the inferior frontal gyrus. Responsibility for decisions affects escalation decisions through recruitment of the superior (inferior) gyrus, when the decision is framed positively (negatively).

## Background

Decision escalation, also called escalation of commitment, refers to a common sunk cost bias in decision making, whereby the decision maker takes into account irrelevant prior information regarding an investment (time, money and/or effort) and consequent emotions, for making future decisions [1]. Under such circumstances, the mere fact that an investment was made increases the likelihood of further investment, even though it may not be the optimal decision. For example, people may continue investing in a failed project (or stand in a long line at a store), even though the best path forward may be to quit the project (or move to a different line), just because they have already invested in the project (or already spent time standing in one line) [2]. This decision escalation effect is prevalent, for example, 54% of consumers in one experiment chose a trip based on sunk cost and not its utility [3].

This phenomenon has two characteristics that reflect decision biases deviating from the economic rationality assumption. The first is the decision bias such that action decisions are more consistent with prior choices, even when other information may suggest alternative optimal paths. The second is the violation of the principle of stochastic dominance. When one choice never pays less and can stochastically pay more than a second option, it is stochastically dominant. Two studies have shown that sunk costs can increase the chance of violating this principle when the expected value is low [4]. Whyte [5] proposed a gain–loss paradigm to explain decision bias. When the decision frame is negative (i.e., a troubled project), the decision to commit further resources is framed as a choice between losses: a sure loss or a possibility of a larger loss with a chance to return to the reference point. Prospect theory suggests that the decision maker will show risk-taking behavior to avoid a sure loss (i.e., escalation is preferred). Such risk-taking behavior may rely on intuitive processing, rather than on analytical processing. Therefore, some studies have argued that escalation bias can be overcome by deliberate thinking [6, 7]. Montealegre and Keil [8] proposed a four-phase de-escalation process that used a systematic procedure for conscious deliberation to make decisions. Based on such insights, it seems that the analytical processing system, slower and more logical, may mediate de-escalation decisions, while the intuitive, gut-feeling-based processing system, faster and more automated, may mediate escalation decisions.

Prior research in sunk costs has reported that the effect exists across different species (such as rats) and is evolutionary [e.g., 9, 10]. Certain neural mechanisms have been identified to be associated with sunk costs. Specifically, it was found that different networks are sensitive to the sunk cost (already invested) amount, and to the incremental (follow-up) cost needed for saving the initial investment [11]. The former network includes regions involved in risk-assessment, such as the bilateral medial and superior frontal gyri. The latter includes regions involved in reward processing, including the caudate nucleus, and regions involved in conflict monitoring, such as the cingulate gyrus. Haller and Schwabe [12] found that reduced activity in the ventromedial prefrontal cortex (vmPFC) and associated increased activity in the dorsolateral prefrontal cortex (dlPFC), presumably representing deficient integration of emotions into decision processes [13], is associated with a larger decision escalation bias. Fujino et al. [14] found that the insula, inferior frontal gyrus (IFG), and anterior cingulate cortex (ACC) are activated during decision escalation.

Together, these studies demonstrate that decision escalation can be mediated by activity in regions involved in risk aversion states, reward processing, integration of emotions and reflections, self-perception and conflict monitoring [11, 12, 14]. They also show that different types of decision escalation (e.g., project-continuum paradigm vs. choosing between two alternative sunk costs, see [14], amounts of sunk cost and required follow-up investment, see [11], can produce different activation patterns involving different brain regions from the broader abovementioned network of regions. If cognitive processing is to overcome decision-making bias, the neural mechanisms of escalation and de-escalation would be different and may also be affected by different contextual factors. More specifically, we anticipate that brain regions associated with intuitive thinking (system 1) may be more activated when decisions are escalated, while brain regions associated with deliberate thinking (system 2) may be more activated when decisions are de-escalated.

Although extant literature has reported certain neural mechanisms associated with sunk costs, there is a key issue that has not been well-studied. That is, what are the neural mechanisms that result in different escalation or de-escalation decisions, and what contextual factors may affect these neural activities? Without such knowledge, it would be difficult to overcome this decision bias. The knowledge has also clinical significance, because decision escalation bias may be accentuated in people with impulse control disorders [15] including for instance, in gamblers [16].

We hence seek to expand current knowledge of decision escalation, and specifically regarding the brain regions that are recruited for escalation and de-escalation decision. We also investigate the effects of two contextual variables: responsibility and framing conditions. It is important to consider escalation and de-escalation decisions independently, because different brain mechanisms may be involved and can encode rewarding and punishing outcomes differently [9, 17]. Responsibility and framing are also two common factors that are used to interpret decision escalation from the perspectives of self-justification theory and approach-avoidance theory, respectively [1, 18–20].

Self-justification theory argues that when the person who made the prior decision may feel that s/he is responsible for the negative outcome and consequently tend to commit more resource to the troubled prior project in order to rectify past losses and attempt to justify his/her earlier decisions [1]. Thus, one’s responsibility for a project is highly related to his/her escalation decisions. In addition, approach-avoidance theory argues that when a goal has both positive and negative aspects and conflicts, the stronger of the two will win [21]. Escalation decisions usually occur when the drive to encourage persistence (i.e. the reward for goal attainment) seems to be greater than the restraint to encourage abandonment (i.e. the punishment for lost) [18]. Therefore, framing of the expected outcome (called goal framing) aims to focus one’s attention on positive or negative consequence of a decision, which will influence one’s escalation decisions or de-escalation decisions [22]. Goal framing is different from other types of framing such as risky choice framing and attribute framing, and would have different effects on decision making. See Levin et al. [22] for details.

Scenarios for decision escalation typically include failed prior projects that need further resource investment. The decision maker may choose to save the project (escalate) or to stop it (de-escalate). Here, we extend previous research on decision escalation in two directions. First, we demonstrate differences between brain networks that govern escalation vs. de-escalation decisions. Second, we account for the role of two contextual factors in affecting sunk cost bias: (1) the framing of information regarding the potential outcome as success/the reward for goal attainment or failure/the punishment for goal failure of the follow-up investment (50% chance to succeed/promotion vs. 50% chance to fail/demotion), and (2) responsibility of the decision maker (whether the initial failed decision is made by the decision maker or others).

In summary, the paradigm of decision escalation is “when decision makers face a prior failed decision and possible outcomes of continuing the project, would they decide to continue or not?” Five theoretical arguments are employed in this research to explain behaviors at different stages of decision escalation and associated neural mechanisms: (1) When decision makers face a failed prior project, their tendency is to escalate the decision in order to revert the situation. This is in line with the arguments of prospect theory and sunk cost effect; (2) When decision makers are responsible for a troubled project, the likelihood of escalation increases based on the self-justification theory. In other words, self-responsibility moderates the sunk cost effect reported in prospect theory; and (3) The likelihood of escalation is affected by the framing of possible outcomes of escalation. The likelihood of escalation is lower in negative framing than in positive framing. In other words, framing moderates the sunk cost effect reported in the prospect theory. This is supported by the approach-avoidance theory and goal-framing effect. Table 1 shows a summary of these theoretical interpretations and associated hypotheses to be elaborated later.

**Table 1:** Summary of hypotheses

Situation	Theoretical arguments	Hypotheses
*Q1: Whether different brain networks govern escalation and de-escalation decisions?*		
Escalation Decisions	Prospect theory: Risk seeking (committing more resources) for lose aversion when receiving negative feedback/facing a troubled project [55] Sunk costs: Committing more resources to save prior investment [3]	Neural areas associated with conflict monitoring, self-perception processes, and emotional processing such as the ACC, cingulate cortex, insula and precuneus will be higher in escalation decisions when a person decides to risk further investment in order to avoid cognitive dissonance and restore his or her self-image. (H1)
De-escalation Decisions	De-escalation process: Using a systematic procedure for conscious deliberation to avoid decision bias [8]	Regions associated with system 2 involved in the inhibition of risky suboptimal choices and learning, namely the inferior and superior frontal gyri will be relatively more active. (H2)
*Q2: What are the effects of responsibility and goal framing on escalation decision?*		
High Responsibility	Self-justification theory: Escalation decisions for rectifying past losses and attempting to justify earlier decisions when one was responsible for the project or made the prior decision [1]	Brain regions associated with escalation decisions will be more activated when one’s responsibility is higher compared to when it is lower (H3a)
Low Responsibility	Self-justification theory: De-escalation decisions occur when one was not responsible for the project and did not make the prior decision [1]	Brain regions associated with de-escalation decisions will be more activated when one’s responsibility is lower compared to when it is higher (H3b)
Positive Framing	Approach-avoidance theory: Escalation decisions occur when the drive to encourage escalation is greater than the restraint to encourage de-escalation. [18] Goal framing effect: Escalation decisions occur when the consequence of escalation behavior is positively framed as gain. [22]	Brain regions associated with escalation decisions will be more activated in positive framing conditions than in negative framing conditions (H4a)
Negative Framing	Approach-avoidance theory: De-escalation decisions occur when the drive to encourage escalation is smaller than the restraint to encourage de-escalation. [18] Goal framing effect: De-escalation decisions occur when the consequence of escalation behavior is negatively framed as loss. [22]	Brain regions associated with de-escalation decisions will be more activated in negative framing conditions than in positive framing conditions (H4b)

A 2 × 2 experiment was designed and conducted using the functional Magnetic Resonance Imaging (fMRI) instrument to explore different neural mechanisms involved in escalation and de-escalation, and how contextual scenarios affect neural processes. The scenarios include two steps: (1) a failed project and (2) potential future outcomes. In prior neuroimaging research on decision escalation, responsibility and framing were mostly constant. They can, however, vary between decision situations. It is therefore important to study their roles, and how they affect the recruitment of brain regions for the escalation and de-escalation decisions. The manipulation of future outcomes (positively versus negatively framed) allows the expected gain–loss to come into the experiment. The approach-avoidance theory suggests that decision makers would prefer options of gains (rewards) to those of losses (punishment). The goal framing indicate that the framing of potential future outcomes may affect decision escalation.

Because negative information from a troubled project may conflict with a decision maker’s existing belief, decisions to escalate failed investments may involve consonance restoration (i.e., trying to ensure that one’s prior decisions, perceptions and future actions are consistent with one another), emotion processing and attempts to save one’s self-image [3, 23–25]. It is reasonable to hypothesize that neural areas associated with consonance restoration and emotional processing such as the ACC, cingulate cortex, insula and precuneus will be higher in escalation decisions (H1).

The cingulate cortex is involved in conflict monitoring, integration of monetary rewards with motor responses, and connecting emotion and memories [26–29]; the insula mediates interoceptive awareness processes and serves as a repository for negative emotions and events [30–33]; and the precuneus mediates self-perception processes [34]. All of these processes are expected to be activated when a person decides to risk further investment in order to avoid cognitive dissonance and restore his or her self-image.

In contrast, when a person decides to cut his or her losses, we posit that decisions become more reflection- and inhibition-dependent. They are consequently likely to involve more momentary risk aversion and the mobilization of inhibition efforts to take the avoidance strategy. We therefore expect that in de-escalation decisions, regions associated with system 2 involved in the inhibition of risky suboptimal choices and learning, namely the inferior and superior frontal gyri [35–37], will be relatively more active (H2).^1^

Given the central role of responsibility in motivating continued investment in failed projects [3, 23], we expect that the abovementioned activations in escalation decisions (in the cingulate cortex, insula and precuneus) will be augmented when one’s responsibility is higher compared to when it is lower (H3a). In other words, when one feels more responsible for the failed investment, stronger mental-self considerations are expected [38] and stronger mobilization of self-image and consonance restoration efforts will be needed. We also expect that when de-escalation decisions are made, low responsibility for past investment should further motivate momentary emphasis on risk aversion. Consequently, inferior and superior frontal gyri are expected to be more activated when the focus that one takes is more on risk aversion than on self- and social-image restoration. We hence anticipate that the expected increased activation in the inferior and superior frontal gyri in de-escalation decisions will be stronger under low responsibility compared to high responsibility conditions (H3b).

Lastly, the framing of potential outcomes can lead to more approach (avoidance) decisions when the provided information is positively (negatively) framed as gain (loss) [18, 22]. It is therefore reasonable to expect that the framing of the success probabilities of the follow-up investment can modulate the effects hypothesized in H1 and H2. We expect that when positive framing is used, a stronger tendency toward escalation decisions (“approach”) will form, and an increase in the associated activity in the regions described in H1 (ACC, insula, cingulate gyrus and precuneus) will be observed (H4a). Similarly, we expect that when negative framing is used, a stronger tendency toward de-escalation decisions (“avoidance”) will form, and an increase in the associated activity in the regions described in H2 (frontal gyri) will be observed (H4b). Table 1 summarizes all hypotheses.

## Results

### Behavioral results

For descriptive purposes, we observe that (1) the overall percentage of escalation decisions and de-escalation decisions were 79.6% and 20.4%, respectively. The percentage of escalation decision was significantly higher than the expected mean value of 0.5 (t = 41.82, p = 0.000); and (2) the time for making escalation decisions (*M* = 1.21, *SD* = 0.23) was significantly shorter (*t* = -3.59, *p* = 0.001) than that for de-escalation decisions (*M* = 1.55, *SD* = 0.57). Table 2 shows the percentage of escalation and the decision time of different scenarios. On average, escalation consumed less decision time than de-escalation. RM-ANOVA revealed that the main effect of framing on escalation decisions was significant (positive framing > negative framing; *F* = 11.612, *p* = 0.002), while the main effect of responsibility was not (*F* = 0.319, *p* = 0.577). The main effects indicate that there is no statistically significant difference in escalation behavior between low and high responsibility alone (main effect), but the potential outcomes framed as gains can lead to more escalation decisions than the potential outcomes framed as losses. Moreover, the interaction effect of these two factors was also significant (*F* = 26.165, *p* = 0.000). This means, responsibility alone does not make significant difference, but framing alone and different combinations of framing and responsibility do make differences. In general, the interaction effect needs further analysis when it is significant.

**Table 2:** The percentage of escalation decision and decision time for each decision type

	High Resp.		Low Resp.		Overall	
	Escalation	De-Escalation	Escalation	De-Escalation	Escalation	De-Escalation
*Escalation (%)*						
Positive	98.4%	1.6%	74.2%	15.8%	86.3%	13.6%
Negative	64.3%	35.7%	81.3%	18.7%	72.9%	27.1%
*Decision time (s)*						
Positive	1.087	1.119	1.240	1.592	1.138	1.561
Negative	1.435	1.572	1.218	1.374	1.290	1.485

**Fig. 1:**
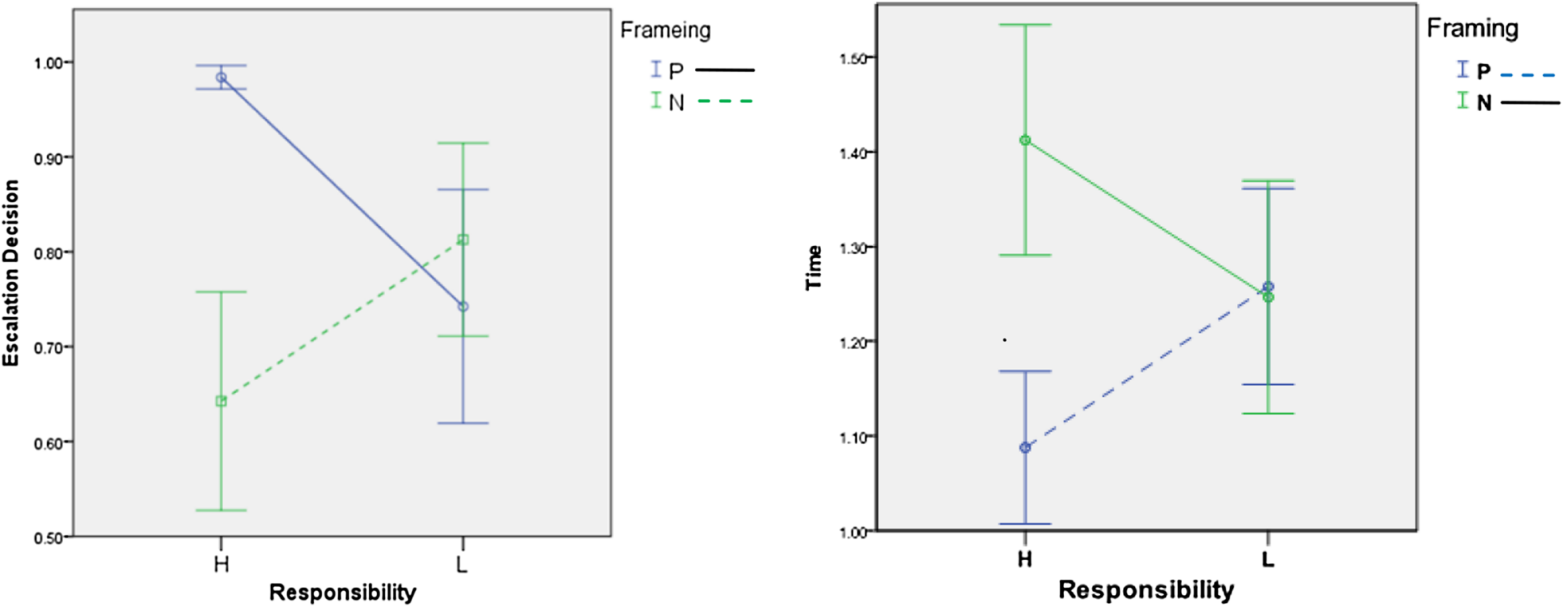
Graph of interaction effect of responsibility and framing on Escalation decision and decision time

Follow-up t-tests on the interaction effect showed that high responsibility conditions increased escalation decisions only when they were positively framed. Unlike expected, high responsibility made it easier for participants to abort the project under negative framing condition. It was marginally significant. Figure 1 shows the escalation percentage and decision time under different scenarios. Tables 3 and 4 show the result of statistical testing.

**Table 3:** Results of RM-ANOVA for escalation decisions and decision time

Source	Escalation decision			Decision time		
	*F* Statistic	Sig.	Eta squared	*F* Statistic	Sig.	Eta squared
Responsibility	0.319	0.577	0.011	0.003	0.959	0.000
Framing	11.612	0.002**	0.293	32.162	0.000***	0.535
Resp. × Framing	26.165	0.000***	0.483	40.408	0.000***	0.591

*p < 0.05

**p < 0.01

***p < 0.001

**Table 4:** Results of paired t-tests for escalation decisions

Condition	Escalation decision			
	Mean	Std.	t	Sig.
High Resp.				
Positive	0.98	0.03	6.212	0.000***
Negative	0.64	0.30		
Low Resp.				
Positive	0.74	0.32	− 1.211	0.236
Negative	0.81	0.27		
Positive framing				
High Resp.	0.98	0.03	3.972	0.000***
Low Resp.	0.74	0.32		
Negative framing				
High Resp.	0.64	0.30	− 1.966	0.059^△^
Low Resp.	0.81	0.27		

^△^*p* < 0.10

*p < 0.05

**p < 0.01

***p < 0.001

### FMRI imaging results

The objective of the analyses below was to address the following four hypotheses on neural association with decision escalation:

H1: activity in the ACC, cingulate cortex, insula and precuneus is higher in escalation decisions.

H2: the inferior and superior frontal gyri are relatively more active in de-escalation decisions.

H3: (a) the abovementioned activations in escalation decisions are augmented in high responsibility conditions, and (b) the abovementioned activations in de-escalation decisions are augmented in low responsibility conditions; and

H4: (a) in positive framing conditions there is stronger activation in the ACC, insula, cingulate gyrus and precuneus; and (b) in negative framing conditions there is stronger activation in the inferior and superior frontal gyri.

**Fig. 2:**
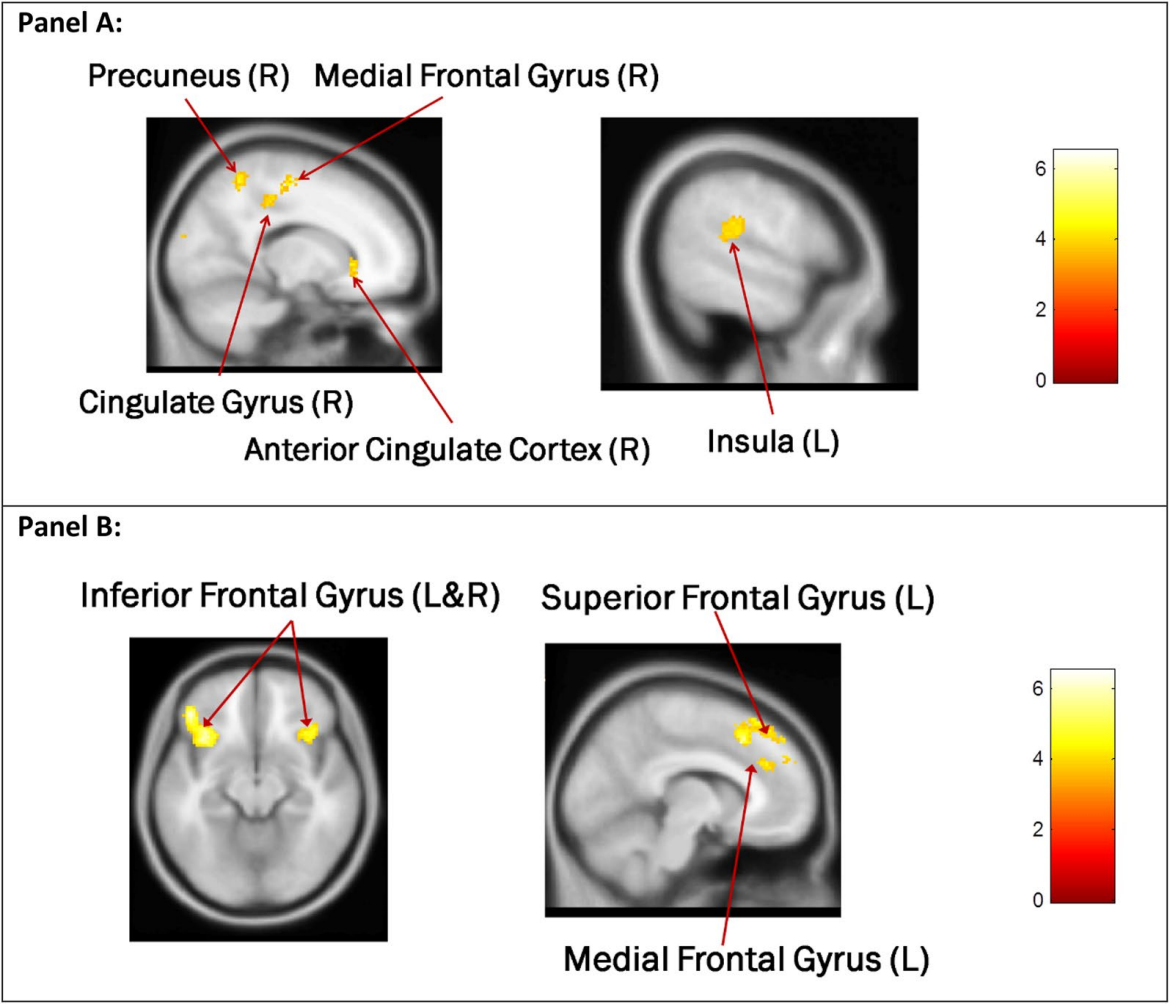
**Panel A:** Regions showing greater activation in escalation decisions than in de-escalation decisions [*P* < 0.001, corrected (False Discovery Rate), cluster size > 153, side-bar represents t-statistics]. **Panel B:** Regions showing greater activation in de-escalation decisions than in escalation decisions [*P* < 0.001, corrected (False Discovery Rate), cluster size > 212, side-bar represents t-statistics]

A whole-brain analysis was conducted to find brain regions associated with escalation decisions and de-escalation decisions. It revealed that the right anterior cingulate cortex (ACC), right cingulate cortex, left insula, right medial frontal gyrus (MFG), and right precuneus were more active in escalation decisions (See Fig. 2, Panel A and Table 5), while bilateral inferior frontal gyrus (IFG), left medial frontal gyrus (MFG), and left superior frontal gyrus (SFG) were more active in de-escalation decisions (See Fig. 2, Panel B and Table 5). Therefore, hypotheses 1 and 2 were supported. Both escalation and de-escalation decisions usually involve using analytical processes or intuitive processes to solve the problems under uncertainty or ambiguous situations. Cingulate cortex and insula activated in escalation decisions involve emotional stimuli, while inferior frontal gyrus and superior frontal gyrus activated de-escalation involve cognitive stimuli. Obviously, de-escalation decisions are more likely to be related to rational processing than escalation decisions. The test result of response time in Table 2 also confirmed this. The decision time for de-escalation decision was longer than that for escalation decision, because rational processing usually need more time to elaborate information.

**Table 5:** Peak cluster activation for Escalation > De-Escalation and De-Escalation > Escalation contrasts

Brain region	MNI coordinates			t-value	cluster size
	x	y	z		
Escalation > De-Escalation					
R. ACC	2	18	− 2	3.94	54
R. Cingulate Gryus	12	− 36	42	4.22	71
L. Insula	− 54	− 32	18	4.13	29
R. Medial Frontal Gyrus	14	− 20	56	5.32	80
R. Precuneus	16	− 54	58	4.69	81
De-Escalation > Escalation					
L. Inferior Frontal Gyrus	− 36	18	− 12	5.74	550
R. Inferior Frontal Gyrus	34	24	− 10	5.26	199
L. Medial Frontal Gyrus	− 6	20	48	6.16	255
L. Superior Frontal Gyrus	− 4	32	50	4.77	285

To test hypotheses on the effect of responsibility (3a-b) and framing (4a-b), ROI masks were created from the WFU PickAtlas Standard Atlases. The contrast analysis, including [High Responsibility > Low Responsibility], [Low Responsibility > High Responsibility], [Positive Framing > Negative Framing], and [Negative Framing > Positive Framing], were conducted. Results in Tables 6 and 7 showed that no significant activations were found in ACC_R, Cingulate Gryus_R, Insula_L and Precuneus_R for [High Responsibility > Low Responsibility] contrast, and in IFG_R, IFG_L, and SFG_L for [Low Responsibility > High Responsibility] contrast. Therefore, H3a and H3b were not supported. This result indicates that responsibility alone does not have significant impact on whether the decision is escalated or not. It is consistent with our behavioral finding that the main effect of responsibility was insignificant. Regarding brain areas associated with framing, positive framing strengthened the activations in ACC_R, insula_L, and precuneus_R, and weakened IFG_L activation. Therefore, H4a and H4b were partially supported when the outcome framing was positive.

**Table 6:** Results of ROI analyses for brain regions hypothesized to be involved in escalation decisions

Brain regions related to Escalation	Responsibility			Framing		
	High > Low			Positive > Negative		
	Contrast value	t	Sig.	Contrast value	t	Sig.
R. ACC	− 0.007	− 0.560	0.710	0.272	1.580	0.063^△^
R. Cingulate Gryus	− 0.224	− 2.088	0.977	0.160	0.979	0.168
L. Insula	− 0.363	− 3.252	0.999	0.314	1.970	0.029*
R. Precuneus	− 0.597	− 3.834	0.999	0.354	1.746	0.046*

^△^*p* < 0.10

*p < 0.05

**p < 0.01

***p < 0.001

**Table 7:** Results of ROI analyses for brain regions hypothesized to be involved in de-escalation decisions

Brain regions related to De-Escalation	Responsibility			Framing		
	Low > High			Negative > Positive		
	Contrast value	t	Sig.	Contrast value	t	Sig.
L. Inferior Frontal Gyrus	0.141	1.283	0.105	0.216	1.335	0.090^△^
R. Inferior Frontal Gyrus	0.120	1.013	0.160	0.112	0.738	0.233
L. Superior Frontal Gyrus	− 0.003	− 0.027	0.510	0.087	0.637	0.265

^△^*p* < 0.10

*p < 0.05

**p < 0.01

***p < 0.001

Because H3a-b was not supported, we further examined the interaction effect of responsibility and framing. Additional contrast analyses were conducted, including [High Responsibility × Positive Framing > Low Responsibility × Positive Framing], [High Responsibility × Negative Framing > Low Responsibility × Negative Framing], [Low Responsibility × Positive Framing > High Responsibility × Positive Framing], and [Low Responsibility × Negative Framing > High Responsibility × Negative Framing]. The results in Tables 8 and 9 showed that there was no significant activation in brain regions hypothesized to be involved in escalation decisions, but activations of all brain regions hypothesized to be involved in de-escalation decisions (i.e. IFG_L, IFG_R, and SFG_L) were weakened by responsibility when messages were framed positively. Therefore, H3b (the inferior and superior frontal gyri are more activated for de-escalation under the low responsibility scenario) was supported under the positive framing condition.

**Table 8:** Results of post Hoc analysis for High Self-Responsibility > Low Self-Responsibility in brain regions hypothesized to be involved in Escalation decisions

Brain regions	Positive framing			Negative framing		
	Contrast value	t	Sig.	Contrast value	t	Sig.
R. ACC	− 0.176	− 1.935	0.990	0.102	1.047	0.152
R. Cingulate Gryus	− 0.179	− 2.509	0.990	− 0.045	− 0.676	0.748
L. Insula	− 0.226	− 3.147	0.998	− 0.137	− 2.049	0.975
R. Precuneus	− 0.395	− 3.965	0.999	− 0.202	− 2.062	0.976

**Table 9:** Results of post Hoc analysis for Low Self-Responsibility > High Self-Responsibility in brain regions hypothesized to be involved in De-Escalation decisions

Brain regions	Positive framing			Negative framing		
	Contrast value	t	Sig.	Contrast value	t	Sig.
L. Inferior Frontal Gyrus	0.446	5.104	0.000***	− 0.304	− 3.555	0.999
R. Inferior Frontal Gyrus	0.257	3.554	0.000***	− 0.137	− 1.584	0.938
L. Superior Frontal Gyrus	0.224	2.520	0.009**	− 0.227	− 2.686	0.994

In order to know whether IFG and SFG mediate the effect on escalation decision, we further performed mediation tests. They showed that the IFG mediated the effect of framing on escalation decisions under the responsibility and negative framing conditions; and that SFG activation mediated the effect of responsibility on escalation decisions under positive framing conditions.

### Post-Hoc mediation analysis

**Fig. 3:**
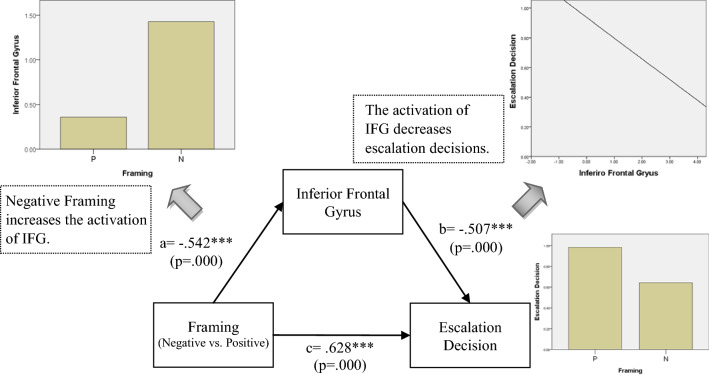
Mediation Model under High Responsibility Condition: framing influences escalation decision through IFG under high responsibility condition

**Fig. 4:**
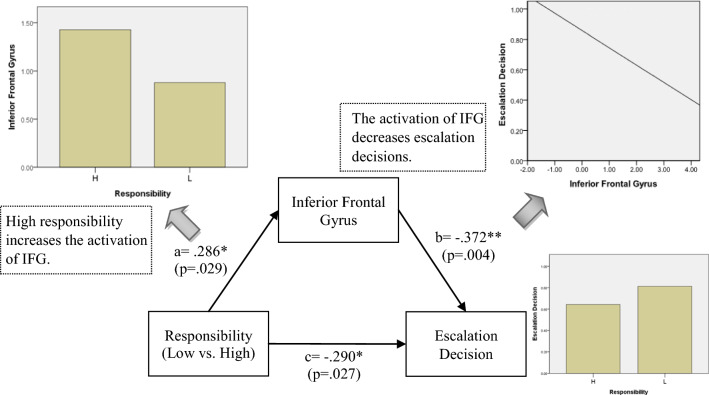
Mediation Model under Negative Framing: responsibility influences escalation decision through IFG under negative framing condition

**Fig. 5:**
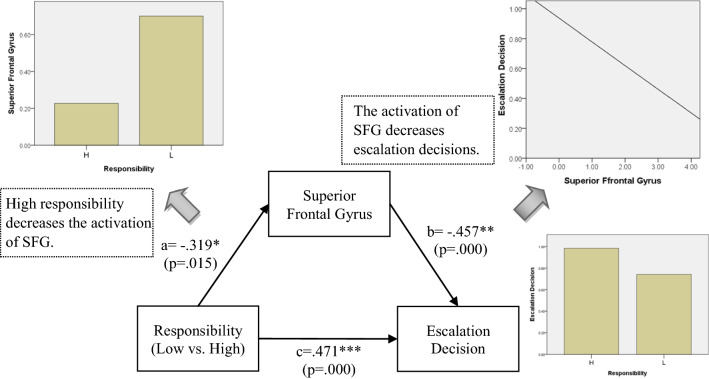
Mediation Model under Positive Framing Condition: responsibility influences escalation decision through SFG under positive framing condition

Mediation models were conducted post-hoc to explore whether the brain activations associated with escalation and de-escalation mediated the relationship between responsibility/framing and escalation decision. As shown in Table 3, there was an interaction effect between responsibility and framing on escalation decision. The mediating role of brain activations, then, was examined for each of three significant conditions in Table 4. The mediation model included treatment (i.e. responsibility or framing) as the predictor, escalation decision as the dependent variable, brain activations from each of the four escalation ROIs and the two de-escalation ROIs as the mediator. Analyses were run using SPSS macro PROCESS with significance determined by 95% confidence interval (CI) based on 1000 bootstrapped samples. The results showed that only the brain regions associated with de-escalation (i.e. IFG and SFG) play mediating role between responsibility/framing and escalation decision (as shown in Figs. 3, 4, 5). First, the IFG mediated the effect of framing on escalation decision under high responsibility condition. When the responsibility is high, negative messages may increase the activation of IFG, and then inhibited the subjects’ escalation decision (see Fig. 3). As shown in Fig. 3, under negative framing condition, the activation of IFG was higher, while the subjects’ escalation decision was lower. Moreover, as the activation of IFG increased, subjects’ escalation decision decreased. This is *why positive messages are more likely to lead to escalation decision than negative messages under high responsibility condition* (As tested in Table 4). In addition, the IFG also mediated the relationship between responsibility and escalation decision under negative framing condition. Responsibility is positively associated with IFG activation, and leads to prohibit the escalation behavior while receiving negative messages (See Fig. 4). As shown in Fig. 4, under high responsibility condition, the activation of IFG was higher, while the subjects’ escalation decision was lower. Moreover, as the activation of IFG increased, subjects’ escalation decision decreased. This explains the marginally significant result in Table 4. That is, *high responsibility would contribute to de-escalation decision under negative framing condition*. Finally, the SFG played a mediation role in the effect of responsibility on escalation decision under positive framing condition. When the subjects received positive messages, *higher responsibility inhibited SFG activation, which resulted in increased escalation decision* (See Fig. 5). As shown in Fig. 5, under high responsibility condition, the activation of SFG was lower, while the subjects’ escalation decision was higher. Moreover, as the activation of SFG decreased, subjects’ escalation decision increased. This finding illustrated the role of SFG in the mechanism of high responsibility on escalation decision under positive framing condition, as shown in Table 4.

## Discussion

This study sought to shed light on (1) differences between the neural underpinnings of escalation and de-escalation decisions, and on (2) how these neural processes may be modulated by key contextual/confounding variables.

The first objective was addressed with H1 and H2. They were supported. The results indicated that escalation decisions engage clusters in the right anterior cingulate gyrus, posterior parts of the cingulate gyrus, precuneus and medial frontal gyrus, as well as a cluster in the left insula. This activation pattern supports the prospect theory perspective that suggests when decision makers face a failed project, they are more likely to avoid losses and choose the risky option (escalation) than maintaining the status quo. That is, escalation decisions require observing the conflict between the choices of accepting the loss and loss recovery (i.e., attempts to overcome past loses, through further or increased investing), and that they are motivated by self-image and interoceptive-awareness [3, 23] as well as by cognitive consonance restoration [39]. In contrast, de-escalation decisions engaged clusters in the bilateral inferior frontal gyrus, and left superior and medial frontal gyri that are more consistent with analytical thinking. This activation pattern supports the postulation that de-escalation requires stronger focus on momentary risk aversion and inhibition [35, 40, 41].

An interesting finding from the study is that the neural mechanisms that lead to escalation or termination of a failed project are different. The former involves more intuitive and emotional decision system (system 1), while the latter involves more of the deliberative system (system 2) [42, 43]. This is also consistent with our behavioral findings that the average time for escalating decisions was shorter than that that for de-escalation. A further question would be whether personality affects individual’s escalation decision. Fujino et al. [14] reported that individuals who tend to adhere to social rules and regulations are more susceptible to the sunk cost effect. Our findings of high responsibility lead to higher escalation is consistent with their finding of personality, as the tendency to adhere to social rules and regulations is more likely to generate higher perceived responsibility. Another issue worth further investigation is whether the risk attitudes of individuals affects their decision escalation. Theoretically, risk takers are more likely to escalate their decisions. Empirical validation, however, will be necessary.

Adding to this, the behavioral results showed that escalation decisions were made significantly faster compared to de-escalation decisions. This supports the assertion that while de-escalation decisions may be associated with more-reflective-analytical mode, escalation decisions are made in a more intuitive mode that focuses on peripheral (e.g., saving self-image) route (system 1) rather than central route (system 2). This view extends extant neuroimaging works on decision escalation after an initial investment [11, 12, 14].

The second objective of the study was addressed the effect of responsibility and outcome framing with H3a-b and H4a-b, respectively. H3a proposed that activation of the cingulate cortex, insula and precuneus will be further increased in escalation decisions, when responsibility is high. H3b suggested that the increased activation of the inferior and superior frontal gyri in de-escalation decisions will be further increased when responsibility is low. Both parts of the hypothesis were not supported. A post-hoc analysis provided partial support for H3b by showing that it may hold true only under positive rather than negative outcome framing conditions. This illuminates the need to account for confounding variables in decision escalation research. These results can be explained by the idea that positive framing is perceived by the decision maker as a potential to gain benefits and hence create additional motivation to escalate the investment, and de-escalation when responsibility was low required additional neural risk aversion and inhibition efforts (i.e., system 2). These efforts are presumed to manifest in increased activation of the frontal gyri. Together, these findings suggest that responsibility, at least in the examined task, is not always confounded in escalation and de-escalation decisions; it becomes relevant only for de-escalation decisions when the success of the follow-up investment is positively framed.

The mediation models contribute to the big picture by showing that while, as expected, different networks are activated in escalation and de-escalation decisions, the switch between such decisions is primarily dependent on inferior and superior frontal gyri regions, which mediate the integration of contextual information, such as framing and responsibility, into escalation vs. de-escalation decisions. This is in line with the functional role of frontal gyri regions in learning and decision making [36, 40].

Previous works have indicated that IFG plays an important role in executive control and inhibiting inappropriate response [44–46]. This is consistent with our mediation analysis results. More specifically, IFG played a mediating role in the relationship between framing and escalation decisions under high responsibility condition (Fig. 3) as well as the relationship between responsibility and escalation decisions under negative framing condition (Fig. 4). In other words, decision makers may reduce the possibility of choosing the risky escalation behavior through the activation of inhibition control mechanism under high responsibility and negative outcome framing. Positive outcome framing, on the other hand, may induce a decision maker’s belief that the project is more likely to succeed [22, 47], which leads to higher activation of the intuitive decision mechanism to escalate the decision.

In summary, the effect of responsibility on escalation reported in previous literature was not supported in our study (the main effect in Table 3). The rationale behind the effect of responsibility is “the greater responsibility one takes, the stronger motivation for rectifying past losses and justifying their earlier decisions would reveal” [1]. That is, decision makers tend to make the decision in a way that is consistent with the prior decision in order to appear rational in their decisions. Arkes and Blumer [3] also reported moderate support that personal involvement increases the sunk cost effect. Our findings do not fully support the argument. Nonetheless, we found that stronger sunk cost effect that leads to escalation decisions when high responsibility and positive outcome expectation are present. The percentage of escalation increases from 74.2% in negative framing to 98.4% in positive framing (see Fig. 1). This conditional finding of responsibility adds new insight into this aspect.

The effect of outcome framing on escalation decision can be explained from the approach-avoidance theory [18]. That is, positive framing provides information of possible gains that decision makers would pursue while negative framing informs possible loss that decision makers tend to avoid. The positive framing focuses attention on the expected gains that makes it easier to self-justify a commitment of more resources (approach) and hence encourages a decision maker to take risks associated with escalation, while the negative framing leads to the other way around (avoidance).

In our study, we found that activation of the cingulate cortex, insula and precuneus increased in escalation decisions (H4a), when the success probabilities of the follow-up investment are positively framed; activation of the inferior and superior frontal gyri increased in de-escalation decisions, when the success probabilities of the follow-up investment are negatively framed (H4b). H4a indicates that brain regions typically associated with system 1 (e.g., ACC and insula) and the midline structure associated with “self” (e.g., precuneus and medial FG) are more activated when decisions are escalated. H4b indicates that brain regions associated with system 2 (e.g., IFG and SFG) are more activated while decisions are de-escalated. The findings support the asymmetry effect of gain–loss, but the activated brain areas are different from those reported in Jessup and O’Doherty [17] whose study reports that brain regions including lateral orbitofrontal cortex, anterior insular cortex, and ACC show an increase in activities to both rewarding and punishing events, while medial frontal cortex and part of ventral striatum responded selectively to the rewarding but not punishing outcomes. This may be due to different experimental settings and also implies that more research is needed. To summarize, *our findings on outcome framing are an important contextual extension of prior research on decision escalation* [11, 12, 14]. We show here that not only framing influences escalation and de-escalation decisions when facing sunk costs, but also expands the separation between the neural activations of brain networks involved in escalation versus de-escalation decisions.

Decision biases occur when objectively equivalent probability is presented as either positive framing or negative framing. As shown in Table 4, negatively framed messages have a lower frequency of escalation biases than positively framed messages under high responsibility condition. In addition, the mediation model showed that negative framing increased IFG activation and further contributed to de-escalation decision. From a practical standpoint, our findings suggest that *decision escalation bias can be alleviated by using more negatively framed success probabilities of follow-up investments, especially under high responsibility condition.* For example, if a person wants to avoid exceeding his or her gambling limit, he or she should think about the probabilities of losing rather than winning the next bet. Similarly, managers should focus on project failure probabilities rather than on success probabilities in order to reduce the risk of being biased by sunk costs. The efficacy of such approaches, though, requires further research. The neural findings further shed light on the brain underpinnings of the shift from image saving to risk aversion focus. This suggests that people with deficits in the abovementioned brain networks may be more (or less) susceptible for decision escalation bias. While some evidence for such effects exists (e.g., it has been shown that gamblers differ from non-gamblers in their follow-up responses to wins and losses, see [48], future research should more closely examine how deficits in any of the brain regions examined here can affect escalation decisions. Future research may also examine the effects of therapies on sunk cost decisions of patients. For example, the ACC tends to be hyper-active in major depressive disorder and in bi-polar disorder subjects; and pharmacological and brain stimulation treatments can reduce ACC activity [49]. The implications of such treatments for decision making in response to sunk costs are unknown, and should be examined.

Another way to attenuate escalation behavior is to decrease the responsibility of decision makers, so as to reduce their discomfort and moderate their tendency to affirm the correctness of their original belief [23, 50]. Low responsibility may help decision makers control and regulate uncomfortable feeling in the pursuit of better results. The result from our mediation analysis (Fig. 5) supports the argument as superior frontal gyrus (SFG) played a mediating role. SFC is a brain area generally believe to correlate with cognitive control, negative feeling regulation, and risk aversion [51–53]. Lower responsibility of a decision maker may lead to avoiding risky behavior by more activation of SFG.

## Limitations

Several limitations of this study are noteworthy. First, the task had fixed success of follow-up investment probabilities, it belonged to the investment-continuum paradigm, and it did not vary the prior investment and follow up costs. Hence, the generalizability of our findings should be extended, by for example, replicating the study while using different decision scenarios, different success probabilities, and different levels of prior and needed investments. Second, the responsibility manipulation did not produce strong neural effects. Different tasks and manipulations may be developed in future research to better elicit such effects. Third, some potential confounding factors such as the forced choice in the experiment and project size (million dollars vs. billion dollars) can be accounted for in future research. Fourth, the decision was targeted at a single decision stage and did not explore the complexity of multi-stage situations; this is a fruitful area for expansion. In addition, we focused on one biasing aspect of sunk cost, and did not delve into nuanced biases, such as the ability of sunk cost to drive violations of the stochastic dominance principle [4]. This also represents an important area for future research. Moreover, inferring value assessment from brain imaging data is difficult [54]. Future studies can use additional experiments to more directly relate value judgments to sunk cost situations. Lastly, this study did not consider attributes of the decision makers, such as personality (especially agreeableness and conscientiousness, see Fujino et al. [14], experience and age of the subjects. Future research may extend our findings by integrating more covariates and predictors into the model.

## Conclusions

Escalation of commitment to a failed project is a common decision bias. The goal of this study was to identify neural correlates associated with the escalation and de-escalation decision and the effect of responsibility and outcome framing. The findings showed that (1) escalation decisions are faster than de-escalation decisions, (2) the corresponding network of brain regions recruited for escalation (anterior cingulate cortex, insula and precuneus) decisions differs from this recruited for de-escalation decisions (inferior and superior frontal gyri), (3) the switch from escalation to de-escalation is primarily frontal gyri dependent, and (4) activation in the anterior cingulate cortex, insula and precuneus were further increased in escalation decisions, when the outcome probabilities of the follow-up investment were positively framed; and activation in the inferior and superior frontal gyri in de-escalation decisions were increased when the outcome probabilities were negatively framed. The findings shed new insight and contribute toward a better understanding of the mechanism underneath the decision escalation.

## Methods

### Study design and procedures

**Fig. 6:**
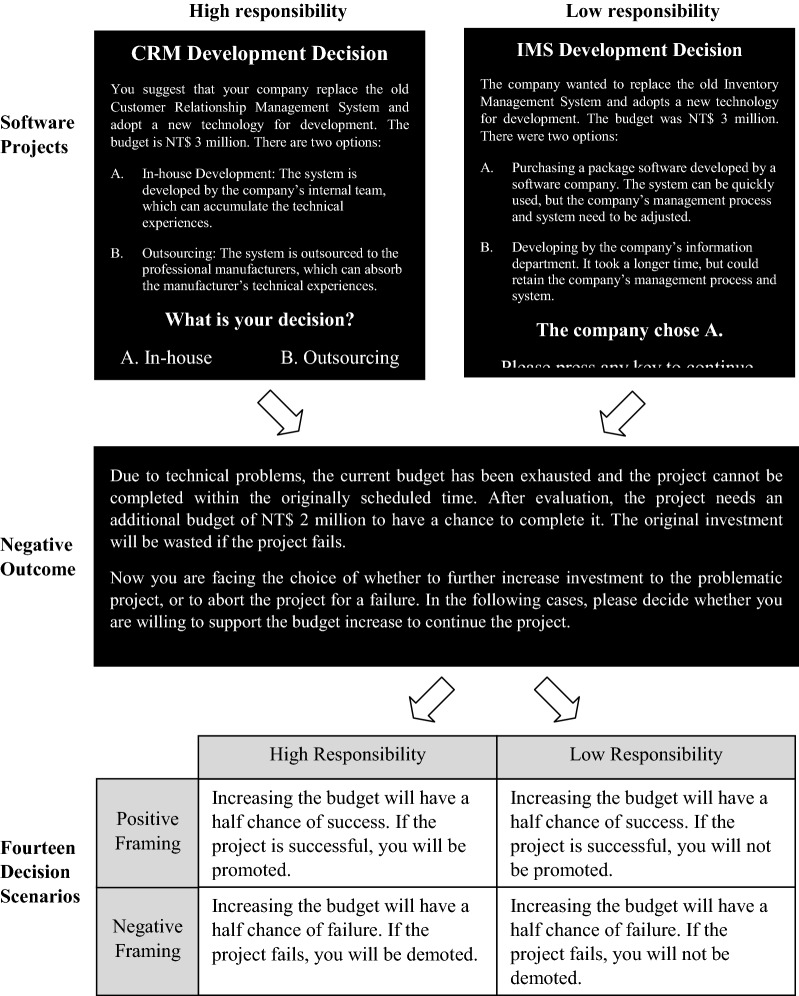
Procedure of the Experiment. Designated experimental scenarios were presented to subjects for their decision. High responsibility indicated asked the subject to commit to a choice, while the low responsibility indicated that the decision was done by others

A 2 × 2 (responsibility x outcome framing) within-subject factorial design was employed. Responsibility was manipulated by presenting four software projects (see Table 10) in which participants were asked to make an initial project decision regarding the development approach (High responsibility condition) and four other projects presented as having the development approach decided by others in the organization (Low responsibility condition), see Fig. 6 for a sample. In addition, the success or failure of the project would be related to the participants under high responsibility condition (decision scenarios presented as “If the project fails, it means you are incapable.”, see Table 11), but not related to the participants under low responsibility (decision scenarios presented as “If the project fails, it doesn’t mean you are incapable.”). Framing was manipulated by presenting decision scenarios with foci on either probabilities of success (Positive framing: “Increasing budget will have a half chance of succeeding with the project") or probabilities of failure (Negative framing: “Increasing budget will have a half chance of failing with the project"). The probability of success and failure was 50%.

**Table 10:** The Projects used in the experiment

No.	Project name	Content presented
1	Customer Relationship Management System Development	You suggest that your company replaces the old Customer Relationship Management System and adopt new technology for development. The budget is NT$ 3 million. There are two options: A. In-house Development: The system is developed by the company’s internal team, which can accumulate the technical experiences B. Outsourcing: The system is outsourced to the professional manufacturers, which can absorb the manufacturer’s technical experiences
2	Database Backup Project	You suggest that your company plans a new database backup solution. The budget is NT 3.1 million. There are two options: A. Use the service provider’s cloud platform for backup. The project takes less time and lower investment costs, but the risk of data leakage is higher B. The backup system is built by the company. The project takes more time and more investment costs, but the risk of data leakage is lower
3	E-commerce Website Building	You suggest that your company builds a new E-commerce website. The budget is NT3.2 million. There are two software development companies bidding: Company A: This company has been established for a long time and has rich experiences in system development, but its system development price is higher Company B: This company has just been established and has less experience in system development, but its system development price is lower
4	Balanced Scorecard Project	You suggest that your company implements a balanced scorecard. The budget is 3.3 million. There are two project manager candidates: Manager A: This manager has extensive project experience but poor communication management skills Manager B: This manager has less project experience but better communication management skills
5	Inventory Management System Development	The company wants to replace the old Inventory Management System and adopt new technology for development. The budget is NT$ 3 million. There are two options: A. Purchasing a package software developed by a software company. The system can be quickly used, but the company’s management process and system need to be adjusted B. Developing by the company’s information department. It took a longer time, but could retain the company’s management process and system
6	Logistics Management Project	The company wants to plan a new logistics management system. The budget is 3.1 million. Need to decide which automatic identification technology to use: A. RFID: Higher cost, but good sensing effect B. QRCode: Lower cost, but poor sensing effect
7	ERP System Project	The company wants to implement a new ERP system. The budget is NT3.2 million. There are two software companies bidding: Company A: This company has more experience in system implementation, but the fees are high Company B: This company has less experience in system implementation, but the fees are low
8	ISO 9001 Quality Management Project	The company wants to implement ISO 9001 Quality Management. The budget is 3.3 million. There are two project manager candidates: Manager A: This manager is a new employee. He/She has a lot of experience in project, but he/she doesn’t know much about the company Manager B: This manager is a senior employee. He/She does not have much experience in project, but has a deep understanding of the company

**Table 11:** The decision scenarios used in the experiment

No.	Decision scenarios
*Positive framing under high responsibility condition*	
P1	Increasing the budget will have a half chance of success. If the project is successful, you will be able to get work achievements
P2	Increasing the budget will have a half chance of success. If the project is successful, it will be able to demonstrate your ability to work
P3	Increasing the budget will have a half chance of success. If the project is successful, it will be able to show your work performance
P4	Increasing the budget will have a half chance of success. If the project is successful, it will be able to prove your excellent judgment
P5	Increasing the budget will have a half chance of success. If the project is successful, it will be able to show your efforts
P6	Increasing the budget will have a half chance of success. If the project is successful, you will be promoted
P7	Increasing the budget will have a half chance of success. If the project is successful, it will be able to show your leadership skills
*Negative framing under high responsibility condition*	
N1	Increasing the budget will have a half chance of failure. If the project fails, it will represent your failure
N2	Increasing the budget will have a half chance of failure. If the project fails, it will show that your work ability is insufficient
N3	Increasing the budget will have a half chance of failure. If the project fails, it will mean you are underperforming
N4	Increasing the budget will have a half chance of failure. If the project fails, it will show that you are inexperienced
N5	Increasing the budget will have a half chance of failure. If the project fails, it will show that you didn’t work hard
N6	Increasing the budget will have a half chance of failure. If the project fails, you will be demoted
N7	Increasing the budget will have a half chance of failure. If the project fails, it will indicate that you have no leadership

### Participants

Twenty-nine participants were recruited with the requirement that they need to have taken at least one Information Systems [IS] course in their college education, 13 females; age range 21–33, *M*_age_ = 23.6). All were healthy, right-handed, experiment naïve, and had normal or corrected-to-normal vision. They had no history of neurological or psychiatric disorders or contraindications to MRI. The experiment was approved by the Research Ethics Committee of National Taiwan University. All participants provided written informed consent and were paid about US$20 for their time.

Participants were asked to take the role of an Information System [IS] manager of a company, in which they are responsible for managing eight software projects that cost a lot of money and need more money to avoid failure. The original investment was sunk cost. It could not be recovered. For each project, they were given 14 decision scenarios in which the projects were in trouble. They had to decide whether to escalate (invest more to save the project) or de-escalate (stop the project).

### MRI procedure

**Fig. 7:**
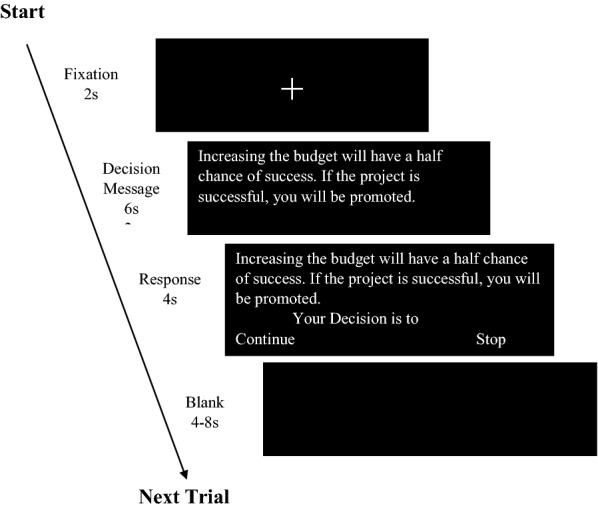
Experimental Paradigm. The problem for requesting an increase of financial commitment was presented to the subject and then ask for the subject to decide whether to stop the project

Before the MRI scanning, participants were given 10 min for reading the descriptions of all eight project scenarios. Then, they were screened for physical and psychiatric disorders. No exclusions were made. Scanning commenced with structural acquisition for anatomic normalization (10 min). Functional scans were acquired from four sessions. In each session, two software projects were randomly assigned (one with high self-responsibility and another with low). Participants were given 20 s to review the description of each software project scenario. In the high self-responsibility condition they were asked to make an initial decision. Next, they performed 14 trials of project decisions in the different manipulated conditions. In each trial, participants were given a decision message for 6 s, followed by a decision response (continuing the project or not) for 4 s. For controlling the clicking movement, the ratio of “continue button” on the left side and the right side was counterbalanced. Each participant performed a total of 112 trials. The experimental paradigm is shown in Fig. 7.

## Data Availability

The datasets used and/or analyzed during the current study are available from the corresponding author on reasonable request.

## Abbreviations

ACC: Anterior Cingulate Cortex
ACC_R: Right Anterior Cingulate Cortex
Cingulate Gyrus_R: Right Cingulate Gryus
dlPFC: Dorsolateral Prefrontal Cortex
fMRI: Functional Magnetic Resonance Imaging
IFG: Inferior Frontal Gyrus
IFG_L: Left Inferior Frontal Gyrus
IFG_R: Right Inferior Frontal Gyrus
Insula_L: Left Insula
IS: Information Systems
M: Mean
MFG: Medial Frontal Gyrus
MNI: Montreal imaging institute
MRI: Magnetic Resonance Imaging
Precuneus_R: Right Precuneus
Resp.: Responsibility
RM-ANOVA: Repeated Measures Analysis of Variance
ROI: Region of interest
SD: Standard Deviation
SFG: Superior Frontal Gyrus
SFG_L: Left Superior Frontal Gyrus
vmPFC: Ventromedial PreFrontal Cortex

## Footnotes

1 Both escalation and de-escalation decisions involve some internal conflict monitoring and processing, but we expect such conflicts to be more pronounced in escalation decisions (H1). The reason is that there is more at stake in such decisions (e.g., double the loss if the project fails after follow up investment) compared to when we accept the smaller loss through de-escalation. Hence, we do not assume significant cingulate cortex involvement in de-escalation decisions.
